# Assessment of Procalcitonin to Predict Outcome in Hypothermia-Treated Patients after Cardiac Arrest

**DOI:** 10.1155/2011/631062

**Published:** 2011-10-26

**Authors:** Pascal Stammet, Yvan Devaux, Francisco Azuaje, Christophe Werer, Christiane Lorang, Georges Gilson, Martin Max

**Affiliations:** ^1^Department of Anaesthesia and Intensive Care, Luxembourg Medical Centre (CHL), 1210 Luxembourg, Luxembourg; ^2^Laboratory of Cardiovascular Research, Public Research Centre for Health (CRP-Sante), 1150 Luxembourg, Luxembourg; ^3^Department of Clinical Biology, Luxembourg Medical Centre (CHL), 1210 Luxembourg, Luxembourg

## Abstract

*Objective*. Determine the potential of procalcitonin (PCT) to predict neurological outcome after hypothermia treatment following cardiac arrest. *Methods*. Retrospective analysis of patient data over a 2-year period. Mortality and neurological outcome of survivors were determined 6 months after cardiac arrest using the Cerebral Performance Category (CPC) score. *Results*. Data from 53 consecutive patients were analyzed. Median age was 63 (54–71) and 79% were male. Twenty-seven patients had good outcome (CPC ≤ 2) whereas 26 had severe neurological sequelae or died (CPC 3–5). At 48 h, after regaining normothermia, PCT was significantly higher in patients with bad outcome compared to those with good outcome: 3.38 (1.10–24.48) versus 0.28 (0–0.75) ng/mL (*P* < 0.001). PCT values correlated with bad neurological outcome (*r* = 0.54, *P* = 0.00004) and predicted outcome with an area under the curve of 0.84 (95% CI 0.73–0.96). A cutoff point of 1 ng/mL provided a sensitivity of 85% and a specificity of 81%. Above a PCT level of 16 ng/mL, no patient regained consciousness. PCT provided an additive value over simplified acute physiology score II. *Conclusions*. PCT might be an ancillary marker for outcome prediction after cardiac arrest treated by induced hypothermia.

## 1. Introduction

Successful cardiopulmonary resuscitation after sudden cardiac arrest (CA) is followed by the early development of a systemic inflammatory response syndrome, characterized by an increase of serum markers of inflammation including C-reactive protein (CRP), tumor-necrosis factor alpha, interleukin-6, and procalcitonin (PCT) [1]. 

In patients with sepsis, PCT has been shown to be a specific marker of bacterial infection, a valuable tool to guide antibiotic therapy, and a predictor of sepsis-associated mortality [2–4]. In contrast, during the early postresuscitation phase following cardiac arrest the diagnostic value of PCT to assess infection is poor [5, 6]. It has been suggested that the increase of PCT is mainly the result of the severity of the postresuscitation disease itself and elevated PCT levels have been associated with an unfavourable outcome in these patients [1, 2, 7, 8].

Mild therapeutic hypothermia (MTH) has been shown to improve neurologic outcome after CA in several studies but may modulate the serum PCT concentration and its utility as an outcome-predictive marker [9–11]. PCT values of 0,5 ng/mL one day after hospitalization were found to be highly predictive for bad neurological outcome in patients after CA without the use of MTH [7]. However, in a second study, PCT values 24 hours after ICU admission varied largely when comparing patients submitted to MTH to normothermic patients [12]. Additionally, no differences between the groups were found when the PCT measurements were repeated 120 hours after ICU admission. They concluded that MTH seems to have a remarkable impact on the inflammatory response after CA. In their study, PCT levels after 24 hours were the single best predictor for bad neurological outcome. However, calculating cutoff values for PCT to predict bad outcome was hampered by the large influence of MTH on PCT serum concentration. To avoid the effect of residual MTH, we performed measurements of serum PCT in patients following cardiac arrest 24 hours after the cessation of hypothermia as a marker for long-term neurological outcome.

## 2. Materials and Methods

This cohort study was conducted in an 18-bed mixed intensive care unit (ICU) of an academic tertiary care centre linked to the national institute for cardiosurgery and interventional cardiology of Luxembourg. This investigation was approved by the National Ethics Committee of Luxembourg and written informed consent from the next of kin of each patient was obtained prior to study inclusion.

All adult patients (age >18 years) with primary cardiac arrest and successful resuscitation with an initial Glasgow coma score (GCS) <8 were eligible for the study. Tracheal intubation, mechanical ventilation, and sedation were mandatory for every patient. Patients with cardiac arrest following trauma, patients with known severe neurological disorders or stroke, head injury, or patients with end-stage noncardiac disease were excluded.

Prior to the transfer to our ICU patients were subjected to coronary intervention according to published international guidelines [13]. Induction of MTH was started immediately after admission to the hospital according to published recommendations for the use of MTH after CA [14]. Briefly, hypothermia was induced at hospital admission using a combination of cold i.v. fluid infusion, surface cooling by ice packs and intravascular cooling (Coolgard with Icy-cath ZOLL Circulation, CA, USA) [15]. Target core (bladder) temperature was 33°C in all patients. After 24 h, rewarming was done actively using the i.v. cooling device at a rate of 0.2°C/h to achieve a core temperature of 36°C at 48 hours after cardiac arrest at the latest.

Arterial blood samples were collected 48 h after cardiac arrest for measurement of procalcitonin (PCT), C-reactive protein (CRP), and white blood cells (WBC). The samples were immediately processed in the central hospital laboratory.

No decision to withdraw or withhold treatment in patients remaining unconscious was taken until sedation was completely stopped for at least 5 days. Thereafter, therapeutic limitations were implemented for patients presenting one or more predefined signs of bad neurological outcome: fixed pupils, seizures, no motor response, absence of somatosensory evoked potentials, and major signs of hypoxic brain damage on CT-Scan or MRI [16].

### 2.1. Data Collection

All pre- and intrahospital data were collected retrospectively from the files of the patients and were recorded according to the Utstein-style template [17]. Only patients with complete datasets were included in the study.

PCT was measured using an electrochimiluminescent immuno assay (ECLIA; B.R.A.H.M.S. AG, Henningdorf, Germany) on the Cobas e601 analyzer (Roche, Basel, Switzerland). A Modular analytics P module (Roche, Basel, Switzerland) was used to determine CRP by immunoturbidimetry using 3rd-generation CRP Tina-quant reagent (Roche, Basel, Switzerland). The functional sensitivity for PCT and CRP testing was 0.09 ng/mL, respectively, 0.6 mg/L. WBC was determined automatically by the Advia 2120 Haematology system (Siemens, Erlangen, Germany). Only values from samples free of haemolysis were used for this study.

Pneumonia was defined as new or progressive infiltrate on the chest X-ray, leucocytosis (>12 000 cells/mm^3^), purulent tracheal aspiration, and microbacterial confirmation of tracheal aspirates.

Sepsis was defined as heart rate >100/min, WBC >12000 cells/mm^3^ or <4000 cells/mm^3^, the clinical evidence or microbiological proof of an infection and hypotension (systolic blood pressure <90 mmHg) with need for vasopressor despite fluid therapy. In contrast to current guidelines, body temperature and respiratory rate were not included in the definition, since all patients were mechanically ventilated and subjected to MTH.

Cardiogenic shock was defined as a systolic blood pressure <90 mmHg despite fluid replacement, the need for inotropes, vasopressors, and the need for intra-aortic balloon pump (IABP) without evidence of infection.

Neurological outcome was assessed by the Cerebral Performance Category (CPC) score by physicians unaware of the blood tests and implicated neither in the treatment of the patient nor in this study [18]. The CPC score classifies patients after CA into 5 categories: CPC 1 (no neurological disability), CPC 2 (minor neurological deficit), CPC 3 (severe neurological impairment, dependent in everyday life), CPC 4 (coma), and CPC 5 (dead). CPC data collected 6 months after cardiac arrest were considered for outcome.

Primary end point of this study was the correlation of PCT values at 48 hours after CA with neurological outcome at 6 months.

Secondary endpoints were the correlation of CRP and WBC values at 48 h after CA with neurological outcome at 6 months.

### 2.2. Data and Statistical Analysis

Continuous variables are expressed as median and interquartile range (IQR) and categorical variables are expressed as count and proportions, unless stated otherwise. All datasets were subjected to the Shapiro-Wilk normality test before statistical analysis. Comparisons between 2 groups of continuous variables were performed using *t*-test for data following a Gaussian distribution and Mann-Whitney test for non-gaussian data. Fisher's exact test was used for categorical variables. Correlation between outcome and PCT, CRP, and WBC was estimated using the Spearman test. The area under the receiver operating characteristic curves (AUC) was used for prediction capacity evaluation. Multiple logistic regression models were implemented to investigate the predictive value of multiple clinical parameters. A *P* value <0.05 was considered significant. 

Statistical analysis was performed using SigmaPlot (Systat Software Inc., CA, USA) and StAR (http://protein.bio.puc.cl/star.html) software.

Sample size calculation reported a minimum number of patients of 30 to detect a significant correlation between outcome and PCT (correlation coefficient 0.5, desired power 0.8, significance threshold 0.05). 

## 3. Results

Between April 2008 and June 2010, 68 consecutive adult patients subjected to MTH after successful cardiopulmonary resuscitation were admitted to our ICU. Five patients died during the first 48 h after admission, nine patients did not have the full dataset available, and one patient had no informed consent signed. Therefore, 53 patients were included in the analysis. The median age was 63 (54–71) years and 42 patients (79%) were male. Cardiac arrest was caused by a cardiac aetiology in 43 (81%), by respiratory failure in 5 (9.5%) and by other nontraumatic causes in 5 (9.5%) patients. The initial rhythm was ventricular fibrillation or pulseless ventricular tachycardia in 35 (66%) patients and asystole or pulseless electric activity in 18 (34%) patients. Out of hospital cardiac arrest (OHCA) occurred in 40 (75%) patients and in hospital cardiac arrest (IHCA) in 13 (25%) patients. 

Patients were dichotomised into two groups according to their CPC score 6 months after CA. Patients were considered having a good neurological outcome when CPC was 1-2 while the patients in the group with bad neurological outcome displayed a CPC score of 3–5.

Demographic and Utstein-style data are presented in Table 1. Bad outcome group patients had a higher SAPS II Score, an increased time to reestablish a sufficient spontaneous circulation and a higher incidence of cardiogenic shock. Eighteen patients in the bad outcome group died after 6 (5–12) days during the ICU stay. At 6 months, 22 out of 26 patients (85%) of the bad outcome group had died (8 (6–19) days after CA) and 4 patients survived with severe neurologic impairment (CPC3). No patient stayed comatose.

The incidence of pneumonia and sepsis was similar in both groups. Nevertheless, antibiotic use was higher in survivors than in nonsurvivors. There was no significant difference in PCT at 48 h in patients with pneumonia versus no pneumonia (0.70 (0–1.71) ng/mL versus 1.30 (0.22–8.31) ng/mL, *P* = 0.15), respectively, sepsis versus no sepsis (1.18 (0.17–78.05) ng/mL versus 0.85 (0.19–3.75) ng/mL, *P* = 0.59).

**Figure 1:**
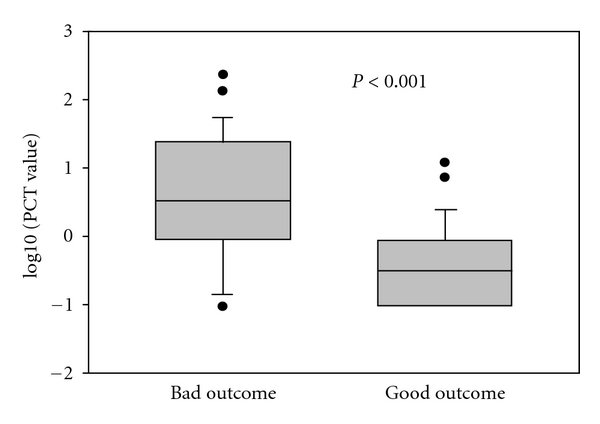
PCT values according to outcome. Patients with bad outcome have significantly higher PCT levels than patients with good outcome. Log10-transformed data are shown.

PCT, CRP, and WBC levels at 48 hours after cardiac arrest are displayed in Table 2. The median PCT value for all the patients was 0.86 (0.19–3.85) ng/mL. Survivors in the good outcome group displayed significantly lower PCT values than patients with bad neurological outcome (Figure 1,  *P* < 0.001). There was a significant correlation between PCT values and bad neurological outcome (*r* = 0.54, *P* = 0.00004).

**Figure 2:**
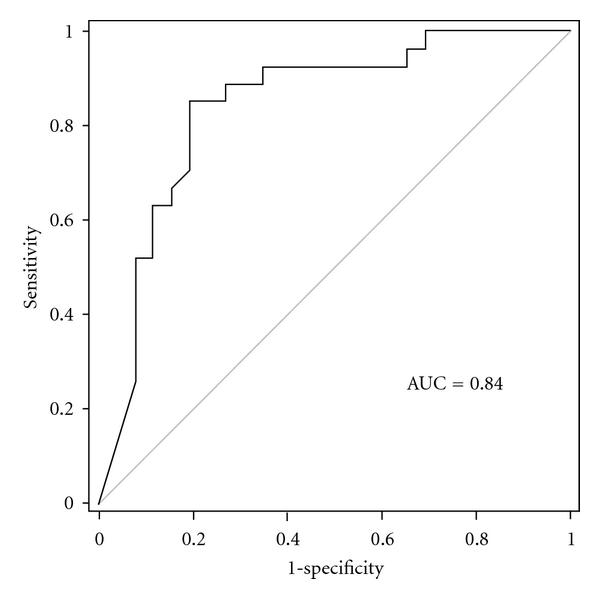
ROC curve showing the significant prognostic value of PCT for neurological outcome.

A receiver operating characteristic curve (ROC) analysis revealed a capacity of PCT to predict neurological outcome with an AUC of 0.84 (95% CI 0.73–0.96; *P* < 0.0001; Figure 2). A cutoff point of 1 ng/mL provided a sensitivity of 85% (95% CI 0.66–0.96) and a specificity of 81% (95% CI 0.61–0.93, FPR 7%). The positive predictive value was 82% and the negative predictive value was 85%. Above a PCT level of 16 ng/mL, no patient regained consciousness, which generates a sensitivity of 100% (95% CI: 0.87–1.00).

No significant correlation was found between CRP and WBC on one hand and PCT on the other hand.

Finally, we investigated the predictive value of PCT in relationship with other clinical parameters such as simplified acute physiology score II (SAPS II), cardiogenic shock, and time to return of spontaneous circulation (ROSC), all known to affect outcome after cardiac arrest. Using multiple logistic regression models, we observed that SAPS II was the best predictor of outcome (*P* < 0.001). PCT as well-predicted outcome (*P* = 0.03), and provided an additive value over SAPS II as showed by an increase of *R* ^2^ values from 0.29 to 0.36 when PCT was added over SAPS II prediction.

## 4. Discussion

The purpose of the present study was to investigate the potential association of serum PCT levels 48 h after successful resuscitation with MTH and neurological outcome after six months in patients following CA. We found increased PCT levels to be a strong predictor of bad neurological outcome with a threshold value of 1 ng/mL providing a specificity of 84%. At a level of 16 ng/mL, PCT predicted bad outcome with a sensitivity of 100%. There was no association between CRP or WBC and neurological outcome.

A marked increase of a variety of proinflammatory cytokines in patients successfully resuscitated after cardiac arrest has been described by several authors. This increase has repeatedly been found to be time-dependent and more pronounced in nonsurvivors than in survivors, indicating the potential utility of these mediators as biomarkers to predict outcome in this patient group [1]. Additionally, biomarkers can be useful to monitor the evolution of systemic inflammation as a consequence of infection. This may apply especially to patients after cardiac arrest. Following successful resuscitation they may require subsequent sedation and mechanical ventilation over a prolonged time period, especially when they are submitted to MTH. Although considered beneficial, hypothermia, and mechanical ventilation may increase the risk to develop pneumonia and other types of infection making an improved surveillance desirable [19, 20].

Recently, procalcitonin has been shown to be a valuable marker to detect bacterial infection, guide antibiotic therapy, and predict outcome in patients with pneumonia and other causes of severe sepsis [2, 21, 22]. In contrast, procalcitonin is only a poor predictor of bacterial pneumonia in patients after cardiac arrest due to a pronounced but unspecific increase of its serum level in the early course of postresuscitation inflammation. In a study by Mongardon et al. 86 out of 132 included patients developed pneumonia [5]. During the first three days, PCT values in these patients were significantly higher than in those without pneumonia. However, using a threshold of 0.5 ng/mL the positive predictive value at day 1 was only 68%, making PCT a rather poor predictor for infection in this setting. Similar results were reported by Schuetz et al. [6]. In a retrospective study on 35 patients after cardiac arrest they found PCT to peak without any statistical difference during the first 24 hours in all patients with or without pneumonia. In contrast, in a earlier study by Oppert et al. the authors observed that an increase of PCT can indicate the onset of ventilator-associated pneumonia, using a cutoff value of 1 ng/mL during the first seven days [23]. In our study we did not investigate specifically the ability of PCT to indicate the onset of infection. However, we did not find any difference of serum PCT levels between patients with and without sepsis or pneumonia.

When interpreting the results of the different studies, several aspects should be taken into account. First, the potential cutoff value of PCT to indicate nosocomial pneumonia lies in exactly the same range of the PCT values observed in patients after cardiac arrest. In a mixed group of ICU patients Charles et al. found a cutoff value of 0.44 ng PCT/mL to detect nosocomial pneumonia at the day of its onset with a specificity of 83.0% [24]. Mean PCT in patients after cardiac arrest but without signs of pneumonia was 0.18 ng/mL (0.11–0.81) at admission and 1.03 ng/mL (0.45–4.68) at day 1 [5]. These data may support doubts that a PCT-based differentiation between infection and postresuscitation inflammation may be possible. Second, combining PCT with other signs of infection or scores to achieve a more precise diagnosis of infection may be difficult since classical signs of infection like fever are abolished by MTH. For the same reason the definitions of sepsis and pneumonia used in our study were only in part applicable. Third, comparison of results from earlier studies may additionally be hampered by a low accuracy of older PCT assays. The assay used in our study has a lower limit of detection of 0,02 ng/mL [25].

Due to its increase after cardiac arrest, PCT has also been tested as a biomarker to predict outcome following successful resuscitation. Most interestingly, PCT has recently been identified not only as a biomarker, but as an active part of the deleterious inflammatory cascade and therefore as a potential therapeutic target [26]. In an earlier study, Fries et al., found a cutoff value of 0,5 ng/mL at day 1 to be predictive for a bad neurological outcome after cardiac arrest in normothermic patients [7]. In a more recent study, the same group investigated the effect of MTH on PCT evolution during the early course of the disease [12]. They observed a strong correlation between bad neurological outcome and increased PCT plasma levels 24 hours after cardiac arrest. This increase was even more pronounced when patients were not submitted to MTH. These data suggest a strong influence of MTH on serum PCT levels making it questionable whether PCT values obtained during hypothermia can be used to predict outcome. In our study, to avoid a potential impact of MTH, PCT was measured 48 hours after cardiac arrest when all patients had regained normothermia. However, additional studies are required to understand the exact influence of MTH on PCT liberation and whether modulation of the inflammatory response could actually be beneficial.

More than just being a marker for neurological outcome, it appears that PCT is more likely to reflect the severity of the ischemic insult eventually resulting in severe organ damage (including the brain) and perhaps death. Therefore, PCT might be a marker of the severity of the postcardiac arrest syndrome [1, 5, 27]. PCT could be a missing link between the neurological tests (clinical examination, electrophysiology, and brain biomarkers) and the general condition of the patient. As PCT broadens the biomarker spectrum to the whole organism, and is not limited to the brain, it could potentially allow a more accurate prognostication of outcome after cardiac arrest [28, 29]. This may also be reflected by significantly higher SAPS II values, a longer time to return of spontaneous circulation, and a higher incidence of cardiogenic shock in the bad outcome group of our study. We further positioned PCT in a multiple logistic regression model as an ancillary marker for outcome prediction when combined with SAPS II. Although SAPS II in itself is a very strong predictor for bad outcome in these patients, addition of PCT may help to even strengthen its value for outcome prediction. PCT must not be used as single outcome predictor but should always be considered in a bundle with other prognostic tools.

Neurological outcome in our study was evaluated at 6 months to assure a stable neurological situation and avoid underestimation of the patients' recovery [30]. In fact, only 4 patients in the bad outcome group survived more than 6 months with persisting severe neurologic impairment. In the studies by Fries and coworkers neurological outcome was tested at day 14 after cardiac arrest [7, 12]. In a third study investigating the predictive value of PCT neurological outcome after CA, hypothermia was only applied in 7 out of 21 patients [8]. No analyses separating patients submitted to hypothermia form those treated with normothermia to evaluate the impact of MTH have been performed. Mean PCT at 12 and 24 hours after CA was significantly higher in patients with unfavourable outcome when compared to the group with a favourable neurological evolution.

Our study clearly has some limitations. As a single centre retrospective trial it has to deal with all inherent risks of bias of this study type. Although a sample size calculation has been done, we must acknowledge that the sample size of our study population might still be too small to be definitively conclusive. Our findings and hypothesis clearly require to be verified in a larger, multicentre and investigator-blinded trial.

We included IHCA and OHCA as well as all types of initial rhythms as we think that a prognostic marker after cardiac arrest should neither be influenced by the location of cardiac arrest nor the initial rhythm to be universally used.

We only studied PCT values at 48 hours after CA, as this was part of the blood sampling protocol of the study. As no PCT values before or beyond this time point were available, we might have missed other time points with significant differences between groups to predict outcome or potential effects of MTH [5, 6].

## 5. Conclusions

In this small study of 53 cardiac arrest patients treated with MTH, we found that PCT, measured 48 hours after cardiac arrest, might be an ancillary predictor for the 6-month outcome after successful resuscitation. A cutoff value above 1 ng/mL predicted bad outcome with an accuracy of 84%. Above 16 ng/mL, PCT predicted bad outcome with a 100% sensitivity (95% CI: 0.87–1.00). Sequential measurements of PCT in larger multicentre trials are required to confirm these findings.

## Figures and Tables

**Table 1:** Main demographic and Utstein data.

	Good outcome (*n* = 27)	Bad outcome (*n* = 26)	*P*
Age (years)	61 (49–68)	67 (59–72)	0.06
SAPS II	59 (52–66)	72 (66–77)	<0.0001
Male (*n*)	23 (85%)	19 (73%)	0.23
OHCA (*n*)	20 (74%)	20 (80%)	0.53
Time to ROSC (min)	20 (10–30)	30 (23–36)	0.005
Time CA to TT (min)	280 (215–340)	285 (176–335)	0.73
Initial rhythm			0.20
VF/VT	23 (85%)	12 (46%)	
Asystole	2 (7.5%)	8 (31%)	
PEA	2 (7.5%)	6 (23%)	
Cardiogenic shock (*n*)	7 (26%)	14 (54%)	0.04
Coronary intervention (*n*)	24 (89%)	21 (81%)	0.41
Pneumonia (*n*)	12 (44%)	8 (31%)	0.30
Sepsis (*n*)	3 (11%)	2 (7.7%)	0.68
Antibiotics treatment (*n*)	25 (93%)	14 (54%)	0.001

SAPS simplified acute physiology score; OHCA out of hospital cardiac arrest; ROSC return of spontaneous circulation; CA cardiac arrest; TT target temperature; VF/VT ventricular fibrillation/ventricular tachycardia; PEA pulseless electric activity.

**Table 2:** Biological parameters at 48 h after CA.

	Good outcome (*n* = 27)	Bad outcome (*n* = 26)	*P*
PCT (ng/mL)	0.27 (0–0.72)	3.7 (0.84–31.65)	<0.001
CRP (mg/L)	94 (79–157)	139 (107–175)	0.12
WBC (cells/mm^3^)	9530 (7235–12935)	12460 (9450–17550)	0.18

PCT procalcitonin; CRP C-reactive protein; WBC white blood cell count.
